# Examining the Influence of Zinc Oxide Nanoparticles and Bulk Zinc Oxide on Rat Brain Functions: a Comprehensive Neurobehavioral, Antioxidant, Gene Expression, and Histopathological Investigation

**DOI:** 10.1007/s12011-023-04043-x

**Published:** 2024-01-08

**Authors:** Amira A. Goma, Alyaa R. Salama, Hossam G. Tohamy, Rashed R. Rashed, Mustafa Shukry, Sara E. El-Kazaz

**Affiliations:** 1 Department of Animal Husbandry and Animal Wealth Development, Faculty of Veterinary Medicine, Alexandria University, Alexandria, 21944 Egypt; 2 Department of Pathology, Clinical Pathology, Faculty of Veterinary Medicine, Alexandria University, Alexandria, 21944 Egypt; 3 Department of Pathology, Faculty of Veterinary Medicine, Alexandria University, Alexandria, 21944 Egypt; 4 Department of Physiology, Faculty of Veterinary Medicine, Kafrelsheikh University, Kafrelsheikh, 33511 Egypt

**Keywords:** Neurobehavior, Nanoparticles, Oxidative stress, Memory, Brain, Gene expression

## Abstract

The study aimed to assess the impact of zinc oxide nanoparticles (ZnONPs) on rats’ neurobehavior compared to bulk zinc oxide (BZnO). Thirty male Sprague-Dawley rats were randomly assigned to five groups. The control group received Tween 80 (10%), while the ZnONP groups were given ZnONPs at 5 and 10 mg/kg body weight dosages, and the bulk zinc oxide (BZnO) groups received BZnO at the same dosages. Behavioral observations, neurobehavioral examinations, and assessments of brain tissue oxidative markers, neurotransmitter levels, and histopathological changes were performed. The results indicated that ZnONP at a dosage of 5 mg/kg improved general behavior, locomotor activity, memory, and recognition and reduced fearfulness in rats. Conversely, the higher dosage of 10 mg/kg and the bulk form had adverse effects on general behavior, locomotor activity, and learning ability, with the bulk form demonstrating the most severe impact—znONP-5 treatment increased antioxidant enzyme levels and decreased inflammatory markers. BZnO-5 exhibited lower oxidative stress markers, although still higher than BZnO-10. Furthermore, ZnONP-5 and BZnO-5 increased neurotransmitter levels compared to higher dosages. ZnONP-5 upregulated the expression of brain-derived neurotrophic factor (BDNF) mRNA, while BZnO-5 showed increased BDNF mRNA expression and decreased expression of genes related to apoptosis and inflammation. In summary, ZnONPs at 5 mg/kg demonstrated positive effects on rat brain function and behavior, while higher dosages and the bulk form had detrimental effects. In conclusion, the studies emphasized the importance of further assessing various doses and forms of zinc oxide on brain health, highlighting the significance of dosage considerations when using nanomaterials.

**Supplementary Information:**

The online version contains supplementary material available at 10.1007/s12011-023-04043-x.

## Introduction

More recently, nanotechnology has appeared as an up-and-coming and rapidly advancing field of research, potentially revolutionizing various scientific disciplines [1]. Its widespread application in numerous areas of life has made it increasingly prevalent. Nanotechnology primarily focuses on manipulating nanoparticles, which are atomic or molecular clusters portrayed by their minute size, typically less than 100 nm. These nanoparticles are amended versions of fundamental elements, achieved by altering their atomic and molecular properties. One of these essential elements is Zn, which is vital for maintaining the average central nervous system (CNS) performance. Its oxide form, ZnO, is a widely used form of metal oxides as a feed additive as it includes a more significant percentage of zinc and is less toxic than other zinc compounds [1]. Moreover, zinc oxide was previously used to treat epilepsy [2].

Nowadays, metal oxide nanoparticles are manufactured for a diversity of potential uses. One of them is the zinc oxide nanoparticles (ZnONPs), considered one of the best exploited at nano dimensions and have a vast area of utilization such as in electronic, optoelectronics, and as an effective nanocarrier for conventional drugs due to their cost-effectiveness and benefits of being biodegradable and biocompatible [3]. Furthermore, zinc oxide nanoparticles have been reportedly involved in medicinal and agricultural uses [4]. It also has other potential applications, such as therapeutic carriers, biological sensing, gene transfer, nanomedicine discovery, biological labeling, medical implant coatings, electronic sensors, wastewater treatment, and communication [5–7]. Zinc oxide nanoparticles (ZnONP) exhibit excellent biocompatibility, transparency, and high electron mobility [8]. Metal oxide nanoparticles, such as ZnO-NPs, possess a broad spectrum of biological applications and effects [9]. These encompass antioxidant, antibacterial, antitumor, antidiabetic, and anti-inflammatory properties, as well as the ability to enhance wound healing [10]. The wide range of activities can be attributed to zinc’s crucial involvement in enzyme systems, the synthesis of proteins and nucleic acids, hematopoiesis, cell proliferation, tissue regeneration, and neurogenesis [11]. Additionally, zinc plays a role in metabolic and signaling pathways, protecting against ischemia/reperfusion injury in organs like the kidney [12]. Metal oxide nanoparticles were deducted to show better efficacy [13], credited to their ability to pass the blood-brain barrier, alter metabolic profiles, and trace element levels in certain brain regions [14].

Learning and memory are interconnected processes whereby information is stored in the brain and retrieved as needed [15]. Trace elements play crucial roles in various physiological functions of brain biochemistry. These functions encompass the synthesis of neurotransmitters, the maintenance of antioxidant defense mechanisms, the regulation of intracellular redox processes, and the modulation of neural cells [16]. Imbalances, either in the form of deficiency or excess, of these trace elements can lead to disorders in the CNS [17]. For example, zinc deficiency can lead to memory loss, known as Alzheimer’s [18], in humans and animals [19]. Furthermore, zinc can also influence blood-brain-barrier (BBB) permeability [20].

Researchers also indicated that ZnONPs could affect the function of neural cells [21] and biocompatibility [22]. Zinc oxide nanoparticles (ZnONPs) have been found to impact brain monoamine levels (dopamine, norepinephrine, and serotonin) and various ions (Ca2+, Na+, K+, and Zn2+). They have also demonstrated the ability to modulate synaptic transmission in vitro [23]. Furthermore, Yongling et al. [24] have reported that zinc oxide nanoparticles (ZnONPs) can improve depressive-like behavior and cognitive impairment in mice. This effect is believed to be achieved by upregulating neuronal synaptic plasticity and functions. Thus, the precise impact and safety of zinc oxide nanoparticles (ZnONPs) on cognitive function remain unclear. To address this question, the main objective of the current study was to evaluate the impact of zinc oxide nanoparticles (ZnONPs) on brain function and behavior, specifically comparing it to the bulk form of zinc oxide (BZnO).

## Materials and Methods

### Chemicals

Tween 80, a chemical compound, was procured from Alpha Chemika Co. in Mumbai, India. Zinc oxide nanoparticles (ZnONPs) were obtained from Sigma-Aldrich Co., a company based in St. Louis, MO, USA. The bulk form of zinc oxide (BZnO) was purchased from Oxford Lab Chem Co. in Mumbai, India.

### Characterization of the ZnONPs

At Alexandria University in Egypt, TEM analysis was used to assess the morphological properties of ZnONPs. The particles were dissolved in Tween 80 and then sonicated for about 15 min. The dispersion was then deposited on the copper grids for drying and examined in the TEM. The particle diameter was evaluated [25].

### Preparation of ZnONP and BZnO Solutions

ZnONPs and BZnO are white powders prepared freshly by dissolving the required amount in Tween 80 (10%). Then, the solutions were sonicated by vortex vibration for 15 min before use.

### Experimental Design

Thirty male Sprague-Dawley rats weighing 130–150 g (8–9 weeks of age) were sourced from the Medical Research Institute at Alexandria University, Egypt. The rats were kept in wired mesh cages under a natural light-dark cycle. They were provided unlimited food and water access, following their nutritional requirements [26]. The basal diet components are documented in Table 1S of the research conducted by Atta et al. [27].

. The experimental protocol received approval from the Alexandria University Institutional Animal Care and Use Committee (ALEXU-IACUC, 013-2022-12-12\180). Following a 1-week adjustment period, the rats were accidentally allotted into five groups, each consisting of six rats. The control group received Tween 80 (10%). ZnONP groups (ZnONP-5 and ZnONP-10) were treated with ZnONPs ˂ 50 nm by 5 and 10 mg/kg bwt, respectively. BZnO groups (BZnO-5 and BZnO-10) administrated two doses of BZnO, 5 and 10 mg/kg, respectively [28]. Rats in all groups were injected intraperitoneally by 1 ml/rat three times/week for 3 weeks. Throughout the experimental period, rats were behaviorally observed. Following the completion of the treatment period, the rats in each group underwent neurobehavioral assessments individually and in a blinded manner. Each test was conducted on a separate day. Afterward, the rats were humanely euthanized by decapitation. The collected brain tissue was divided into two equal portions for further analysis. In subsequent investigations, the first portion was stored at −80°C to examine oxidative markers and neurotransmitter levels. The second part underwent histological analysis by being preserved in formalin.

### Behavior Analysis

During the 3-week experiment, using focal observation, rat behavior was observed three times a week for 4h daily. The 4h were divided into two during the late morning period (9.00a.m.:12.00p.m.) and another two during the early afternoon (12.00:3.00p.m.). The behaviors observed were ingestion, body care, movement activities, resting, and investigation [29].

### Neurobehavioral Tests

First, the open field test comprises a square area with an inner identified central area. The arena’s black lines separated it into 16 even halves. Rat was allotted to explore the arena for 5 min after being located in the corner of the outer area. The apparatus was wiped utterly after each rat. The parameters evaluated were lines crossed peripherally and/or centrally, rearing frequency, licking and scratching frequencies, freezing time, fecal pellets number, and urination frequency. Anxiety and motor activity were measured with this procedure. Fewer lines crossed and higher rearing frequency indicates weak locomotor activity and increased anxiety [24, 30].

Second, the elevated plus maze (EPM) comprises two open arms measuring 50 × 10 cm and two closed arms of the same size, featuring side walls that are 40 cm high. The edges of the open arms are 0.5 cm high. The arms are connected, forming a 10 × 10-cm central arena. The EPM was atop the floor by about 50 cm. The rat was permitted to investigate the maze for 5 min, starting from the central arena and facing the open arm. The arena was cleaned after each rat. The evaluated parameters were waiting time to enter either arm, time of presence in the open or closed arms, frequency of entry into either arm, percentage of presence time in the open or closed arms, and % of entries in either arm [28]. These parameters determine the anxiety level as longer presence time in the open arms indicates an anxiolytic response. However, a longer time in closed arms reflects an anxiogenic response.

Third, the Morris water maze (MWM) is composed of a circular tank with dimensions of approximately 120 cm in diameter and 60 cm in height. The tank is filled with opaque water, which is made opaque by the addition of non-toxic ink. The water depth is maintained at 45 cm. The container was partitioned into four equal quadrants by two imaginary crossed lines. Quadrant III was chosen as the target quadrant containing the platform (5 cm in diameter), placed below the water by 1–2 cm—the testing procedure comprised two phases: place navigation and spatial probe. During the place navigation phase, each rat was given four trials to locate and reach a hidden platform within the water maze, which measured their learning ability. The rats were placed into the water from different quadrants and allowed to swim for 60 s or until they reached the platform. The trials were conducted in a clockwise manner, and the position of the platform remained consistent throughout all trials. If a rat failed to locate the platform within 60 s, it was manually placed on it for 5 s before being returned to its home cage. A 5-min interval was provided between each trial, and the time taken by each rat to locate the platform in each trial was recorded. In the spatial probe phase, conducted on the second day, the platform was eliminated to assess the rat’s memory and recognition. The rat was released into the water from the quadrant opposite the target quadrant and given 60 s to explore. The time the rat reached the target quadrant, its presence time in the target quadrant, and the number of trials needed to enter the target quadrant were recorded [24].

### Brain Oxidative Markers Analysis

After being homogenized in PBS, brain tissues were prepared to assess oxidative markers. The resulting supernatants were utilized for measuring malondialdehyde (MDA) [31] and catalase (CAT) activity, following the method described by Aebi [32]. This method employs catalase to decompose H2O2. Following a 1-min incubation period, the sample containing catalase and a known concentration of H2O2 is treated with sodium azide to stop the reaction. The remaining hydrogen peroxide (H2O2) was quantified using a reaction involving 4-aminophenazone (AAP), 3,5-dichloro-2-hydroxybenzenesulfonic acid (DHBS), and horseradish peroxidase (HRP). A quinoneimine dye was formed in this process, and its wavelength was determined to be 510 nm. Kits available for purchase were used to measure SOD activity following the protocols established by Nishikimi et al. [33]. Glutathione peroxidase (GPx) activity was determined using a technique pioneered by Paglia and Valentine [34]. Glutathione reductase and NADPH were combined to convert oxidized glutathione (GSSG) to its reduced form efficiently while simultaneously oxidizing NADPH to NADP. The reaction’s advancement was assessed by measuring the reduction in absorbance at 340 nm.

The levels of interleukin-6 (IL-6) and tumor necrosis factor (TNF-α) were gauged through ELISA kits (MBS355410 and MBS282960, respectively) procured from My BioSource, Inc., situated in San Diego, USA. Brain acetylcholinesterase (AChE) activity was assessed colorimetrically using a kit supplied by Sigma-Aldrich in St. Louis, MO, USA. Reactive oxygen species (ROS) levels in the brain tissue homogenate were determined following the protocol outlined in the cited reference [35]. In short, the method entailed transforming 2,7-dichlorofluorescein diacetate into the fluorescent 2,7-dichlorofluorescein in hydrogen peroxide. The fluorescence emission was then assessed at a wavelength of 525 nm using a fluorescence plate reader, with an excitation wavelength of 488 nm. Protein concentrations were determined through the Bradford assay to standardize the biochemical parameters.

### Brain Neurotransmitters Analysis

Acetylcholine levels in the brain supernatants were evaluated using a colorimetric choline/acetylcholine assay kit (BioVision Inc., Waltham, MA, USA, Catalog No. K615) at a wavelength of 570 nm. The assay has a detection range of 10 pmol to 5 nmol for choline/acetylcholine in the samples. Additionally, serotonin and dopamine levels were quantified using competitive ELISA kits (BioVision, Catalog Nos. E4294 and K4219, respectively). The detection range for serotonin measurement was 15.625 to 1000 ng/ml, while for dopamine, it was 1.56 to 100 ng/ml. The detection sensitivity for serotonin was below 9.375 ng/ml, and for dopamine, it was below 0.938 ng/ml.

### RT-PCR

Gene expression in brain tissue was evaluated through quantitative RT-PCR. Total RNA, extracted from about 100 mg of brain tissue, utilized TRIZOL reagents from Invitrogen in Carlsbad, CA, USA. Nanodrop was employed for quantifying RNA samples. A cDNA synthesis kit from Fermentas in Waltham, MA, USA, was employed for DNA synthesis. RNA samples with an A260/A280 ratio of 1.8 or higher were chosen for cDNA synthesis. The resulting cDNA was amplified using SYBR Green Master Mix and specific primers detailed in Table 2S. The housekeeping gene GAPDH served as a reference for normalizing cDNA levels. Data from the amplification were analyzed using the 2^(−ΔΔCt) method [36].

### Histopathological Analysis

The experimental rats’ cerebral and cerebellar brain tissue was gathered and immediately immersed in 10% formalin for a day. Afterward, the fixed brain tissue samples underwent a series of steps, including washing, dehydration with progressively stronger alcohol concentrations, clearing with xylene, and embedding in paraffin wax. Thin sections, approximately five microns thick, were then crafted from the paraffin blocks and subjected to staining with H&E.

### Immunohistochemistry of Caspase-3 Protein and Quantitative Analysis

Immunohistochemistry was performed on paraffin-embedded tissue sections from the cerebrum and cerebellum of treated rats, following the standard horseradish peroxidase (HRP) method. The tissue sections were affixed to positively charged slides. A rabbit anti-rat caspase-3 antibody from Lab Vision was utilized according to the manufacturer’s instructions. The approximately 4-μm thick sections were dewaxed and rehydrated. The sections underwent treatment with 3% H2O2 to inhibit endogenous peroxidase activity. Antigen retrieval was achieved by microwaving the slides in 10 mM sodium citrate buffer (pH 6.0) for 10 min. Following this, the sections were incubated with the primary antibody, washed with Tris buffer saline, and the secondary antibody was applied. Subsequently, the sections were treated with 3,30-diaminobenzidine (DAB) substrate chromogen solution, counterstained with hematoxylin, and observed under a light microscope. To calculate the area percentage of caspase-3-positive brown immunostaining cells, ten different fields at a magnification of ×400 were captured and analyzed using ImageJ software.

### Data Analysis

Statistical analysis was conducted using the Statistical Package for Social Sciences software (SPSS, version 25). One-way analysis of variance (ANOVA) was employed unless otherwise indicated. The findings are presented as means ± standard error, with statistical significance established at *P* < 0.05. A post hoc analysis utilizing Duncan’s multiple range test was conducted to discern treatment variations.

## Results

### ZnONP Characterization

**Fig. 1:**
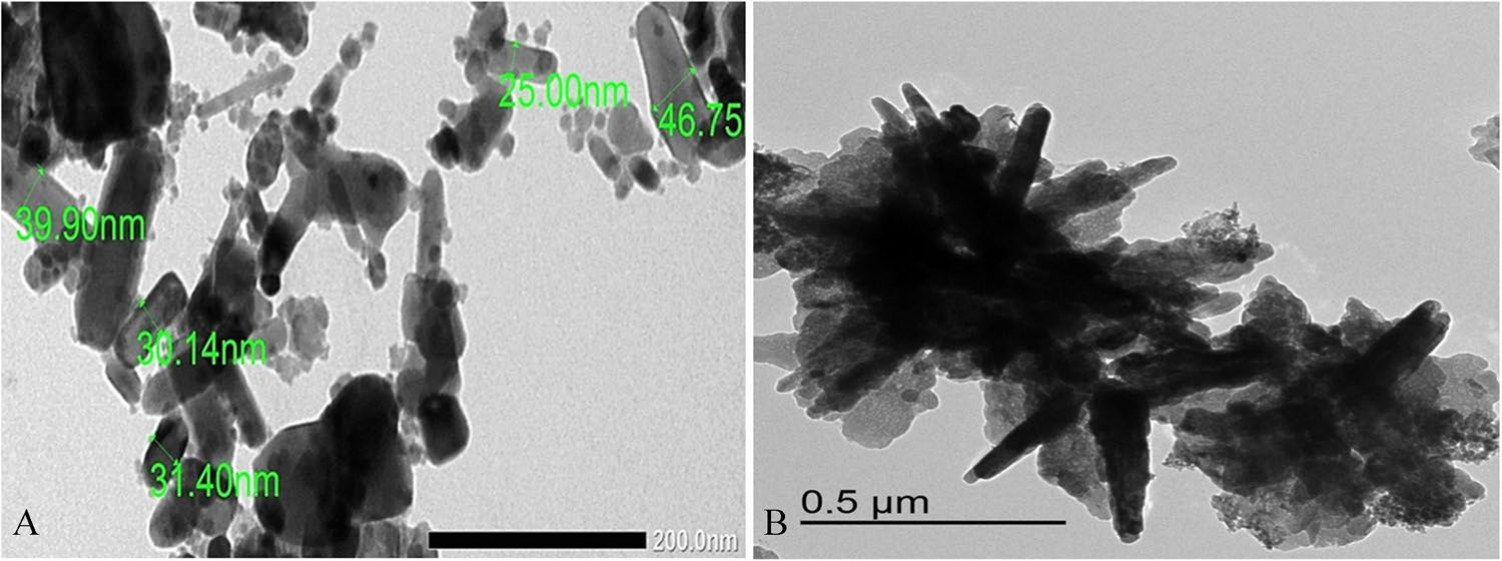
Image by TEM to ZnONP (**A**) indicating the shape and diameter below 50 nm (scale bar = 200 nm), and bulk zinc oxide (**B**)

ZnONP diameter analysis by the transmission electron microscope indicated the polygonal shape of particles with a diameter less than 50 nm, as identified by the manufacturer (Fig. 1).

### Behavioral Observations

The overall behavioral patterns were examined, revealing a noteworthy rise in feeding and standing time, drinking, movement, and total investigation frequencies within the ZnONP-5 group compared to the other groups (*P*<0.0001). Similarly, the BZnO-5 and control groups exhibited a substantial increase in these behaviors compared to the ZnONP-10 and BZnO-10 groups. Conversely, these behaviors experienced a decline in the ZnONP-10 and BZnO-10 groups, with the BZnO-10 group displaying the lowest frequencies among all groups. However, lying time, licking, and scratching frequencies increased in the ZnONP-10 and BZnO-10 groups compared to the other groups, with the BZnO-10 group demonstrating the highest frequencies. A frequency decrease was observed in the BZnO-5 and control groups compared to the ZnONP-10 and BZnO-10 groups. Additionally, a significant reduction was noted in the ZnONP-5 group compared to the other groups (Table 1).

**Table 1:** Effects of different interventions on behavioral patterns of adult male rats

Behaviors	Ingestive		Resting		Body care		Movement activity	Investigation
Treatment	Feeding (min/hr)	Drinking (freq/hr)	Lying (min/hr)	Standing (min/hr)	Licking (freq/hr)	Scratching (freq/hr)	Movement (freq/hr)	Total investigation (freq/hr)
Control	12.50±0.23^b^	7.50±0.23^b^	20.74±0.34^c^	5.26±0.56^b^	2.74±0.30^c^	4.19±0.46^c^	14.39±1.30^b^	2.24±0.20^b^
ZnONP-5	25.98±1.06^a^	8.59±0.52^a^	18.80±0.37^d^	11.67±0.20^a^	1.28±0.17^d^	2.48±0.20^d^	26.00±1.73^a^	3.02±0.41^a^
ZnONP-10	2.89±0.33^c^	1.98±0.23^c^	28.50±0.40^b^	3.65±0.39^c^	3.54±0.36^b^	5.78±0.69^b^	9.11±0.98^c^	1.48±0.20^c^
BZnO-5	11.19±0.29^b^	7.33±0.20^b^	21.09±0.32^c^	5.15±0.57^b^	2.33±0.28^c^	4.22±0.66^c^	16.91±1.30^b^	2.33±0.20^ab^
BZnO-10	1.00±0.21^d^	1.00±0.14^d^	35.70±0.78^a^	2.24±0.25^d^	4.50±0.23^a^	12.00±0.18^a^	5.54±0.63^d^	0.78±0.16^d^

The data were expressed as mean ± SEM, and statistical analysis was performed using one-way analysis of variance (ANOVA) followed by Duncan’s multiple range test for multiple comparisons. In the column, mean values labeled with different letters (^a, b, c, d^) were significantly different at a significance level of *P* < 0.0001

### Neurobehavioral Tests

#### Open Field Test

The locomotor activity in the open field test, shown in Table 2, indicated a decrement in the locomotor activity of the ZnONP-10 and BZnO-10 groups, as there was a decrease in the total number of lines crossed than other groups. In contrast, the ZnONP-5 group had increased locomotor activity, showing more lines crossed than other groups, remarkably the ZnONP-10 and BZnO-10 groups. Additionally, there was a considerable increase in locomotor activity in the BZnO-5 and control groups concerning ZnONP-10 and BZnO-10. Involving anxiety-like behaviors, the ZnONP-10 and BZnO-10 groups were the most anxious groups as they were higher in the central lines crossing, rearing frequency, licking and scratching frequencies, fecal counts, urination number, and freezing time than other groups. The BZnO-10 group had the highest anxiety-like behaviors than other groups. However, the least fearful group was ZnONP-5, as it was lower in all anxiety-like behaviors than other groups, especially ZnONP-10 and BZnO-10 groups. Additionally, there was a significant decrease in anxiety parameters in the BZnO-5 and control concerning ZnONP-10 and BZnO-10 groups. All anxiety-like behavior data of adult male rats are shown in Table 3.

**Table 2:** Impact of various interventions on locomotor activity of adult male rats in the open field test

Measurements	Lines crossed central (*n*)	Lines crossed peripheral (*n*)	Total lines crossed (*n*)
Control	8.83±0.31^c^	60.67±3.11^b^	69.50±2.85^b^
ZnONP-5	4.00±0.73^d^	83.00±1.93^a^	87.00±2.35^a^
ZnONP-10	12.50±0.76^b^	24.67±0.71^c^	37.17±1.17^c^
BZnO-5	8.67±0.42^c^	58.33±2.91^b^	67.00±3.20^b^
BZnO-10	15.67±0.71^a^	17.50±1.38^d^	33.17±0.91^c^

The data were expressed as mean ± SEM, and statistical analysis was performed using one-way analysis of variance (ANOVA) followed by Duncan’s multiple range test for multiple comparisons. In the column, mean values labeled with different letters (^a, b, c, d^) were significantly different at a significance level of *P* < 0.0001

**Table 3:** Impact of different interventions on the anxiety-like behaviors of the adult male rats in the open field test

Measurements	Rearing (freq)	Licking (freq)	Scratching (freq)	Freezing time (sec)	Fecal pellets (*n*)	Urination (freq)
Control	24.00±1.00^c^	6.50±0.76^c^	2.67±0.33^c^	4.50±0.76^c^	9.00±0.58^c^	8.83±0.79^c^
ZnONP-5	18.67±0.49^d^	1.50±0.22^d^	0.83±0.31^d^	2.33±0.33^d^	2.83±0.60^d^	1.50±0.22^d^
ZnONP-10	27.00±1.59^b^	11.67±0.67^b^	5.17±0.48^b^	10.17±0.60^b^	12.00±0.73^b^	12.50±0.76^b^
BZnO-5	22.17±0.95^c^	8.00±0.58^c^	2.17±0.31^c^	6.33±0.88^c^	7.67±0.49^c^	8.33±0.49^c^
BZnO-10	36.67±0.71^a^	14.50±0.76^a^	7.17±0.60^a^	15.50±0.76^a^	15.83±1.01^a^	15.83±0.95^a^

The data were expressed as mean ± SEM, and statistical analysis was performed using one-way analysis of variance (ANOVA) followed by Duncan’s multiple range test for multiple comparisons. In the column, mean values labeled with different letters (^a, b, c, d^) were significantly different at a significance level of *P* < 0.0001

#### Elevated Plus Maze Test

The anxiety-like behavior in the elevated plus maze test (Tables 4, 5) revealed an anxiogenic response in ZnONP-10 and BZnO-10 groups than other groups, with the highest in the BZnO-10 group; this was clear in the longer waiting time to enter either open or closed arms, multiple entries, and more extended presence in the closed arms (*P*<0.0001). However, ZnONP-5 was the more anxiolytic group than other groups, especially ZnONP-10 and BZnO-10 groups, indicated by shorter entry time to open arms, multiple entries in open arms, and longer time of presence. The same anxiolytic response was also present in the BZnO-5 and control groups concerning ZnONP-10 and BZnO-10.

**Table 4:** Impact of different interventions on the performance of adult male rats in the open arms of the elevated plus maze test

Measurements	Waiting time to enter open arms (sec)	Freq of entry into open arms	Time of presence in open arms (sec)	Percentage of presence time in the open arms	Percentage of entries in the open arms
Control	13.50±0.76^c^	15.17±0.70^b^	56.33±0.88^b^	26.70±0.29^b^	56.96±2.30^b^
ZnONP-5	7.33±0.88^d^	21.67±0.67^a^	82.67±0.88^a^	41.25±1.03^a^	88.38±2.02^a^
ZnONP-10	24.33±1.23^b^	7.00±0.58^c^	33.50±1.18^c^	13.74±0.38^c^	24.36±1.77^c^
BZnO-5	15.00±0.58^c^	14.00±0.58^b^	55.83±0.60^b^	26.34±0.29^b^	53.86±1.80^b^
BZnO-10	28.67±0.88^a^	3.00±0.58^d^	24.67±0.88^d^	9.83±0.31^d^	8.69±1.69^d^

The data were expressed as mean ± SEM, and statistical analysis was performed using one-way analysis of variance (ANOVA) followed by Duncan’s multiple range test for multiple comparisons. In the column, mean values labeled with different letters (^a, b, c, d^) were significantly different at a significance level of *P* < 0.0001

**Table 5:** Impact of different interventions on the performance of adult male rats in the closed arms of the elevated plus maze test

Measurements	Waiting time to enter closed arms (sec)	Freq of entry into closed arms	Time of presence in closed arms (sec)	Percentage of presence time in the closed arms	Percentage of entries in the closed-arms
Control	12.50±0.76^c^	11.50±0.76^c^	154.67±1.63^c^	73.30±0.29^c^	43.04±2.30^c^
ZnONP-5	7.50±0.76^d^	2.83±0.48^d^	118.33±5.21^d^	58.75±1.03^d^	11.62±2.02^d^
ZnONP-10	27.33±0.67^b^	21.67±0.67^b^	210.17±1.58^b^	86.26±0.38^b^	75.64±1.77^b^
BZnO-5	13.83±0.48^c^	12.00±0.58^c^	156.17±1.45^c^	74.79±0.09^c^	46.14±1.80^c^
BZnO-10	32.00±0.97^a^	31.50±0.76^a^	226.17±1.72^a^	90.17±0.31^a^	91.31±1.69^a^

The data were expressed as mean ± SEM, and statistical analysis was performed using one-way analysis of variance (ANOVA) followed by Duncan’s multiple range test for multiple comparisons. In the column, mean values labeled with different letters (^a, b, c, d^) were significantly different at a significance level of *P* < 0.0001

#### Morris Water Maze Test

Table 6 displays the learning ability of rats as assessed in the place navigation section of the Morris water maze. The ZnONP-5 group exhibited a significant decrease in locating the hidden platform and reaching the target quadrant associated with the other groups, particularly the ZnONP-10 and BZnO-10 groups. Similarly, there was a significant decrease in the BZnO-5 and control groups compared to the ZnONP-10 and BZnO-10 groups. Conversely, the ZnONP-10 and BZnO-10 groups took longer to locate the hidden platform and reach the target quadrant than the other groups, with the BZnO-10 group displaying the most extended duration.

**Table 6:** Effects of various interventions on the learning ability of adult male rats in the place navigation section of the Morris water maze test

Measurements	Time spent to locate platform (sec)
Control	13.63±0.46^d^
ZnONP-5	4.75±0.46^e^
ZnONP-10	27.54±1.05^b^
BZnO-5	17.54±0.64^c^
BZnO-10	35.79±1.15^a^

The data were expressed as mean ± SEM, and statistical analysis was performed using one-way analysis of variance (ANOVA) followed by Duncan’s multiple range test for multiple comparisons. In the column, mean values labeled with different letters (^a, b, c, d^) were significantly different at a significance level of *P* < 0.0001

Furthermore, the memory and recognition of adult rats measured in the spatial probe section of the Morris water maze are presented in Table 7. The longer time of presence in the objective quadrant and the number of trials to get into the objective quadrant were revealed in the ZnONP-5 group than in other groups. In the same context, there was a substantial increase in the BZnO-5 and control concerning ZnONP-10 and BZnO-10 groups. In contrast, the ZnONP-10 and BZnO-10 groups showed shorter presence times in the objective quadrant and fewer trials to get into the target quadrant than other groups. The BZnO-10 group was the lowest among different groups in the same context.

**Table 7:** Effects of various interventions on the memory and recognition of adult male rats in the spatial probe section of the Morris water maze test

Measurements	Time spent to reach target quadrant (sec)	Time of presence in target quadrant (sec)	Number of trials to get into the target quadrant
Control	12.50±0.76^d^	20.83±0.60^b^	13.50±0.76^b^
ZnONP-5	6.00±0.68^e^	35.67±1.48^a^	17.50±0.76^a^
ZnONP-10	24.50±1.12^b^	11.50±0.76^d^	8.33±0.33^d^
BZnO-5	18.50±0.76^c^	17.33±0.42^c^	11.50±0.76^c^
BZnO-10	32.33±1.93^a^	6.17±0.60^e^	5.17±0.60^e^

The data were expressed as mean ± SEM, and statistical analysis was performed using one-way analysis of variance (ANOVA) followed by Duncan’s multiple range test for multiple comparisons. In the column, mean values labeled with different letters (^a, b, c, d^) were significantly different at a significance level of *P* < 0.0001

### Brain Oxidative Markers

**Fig. 2:**
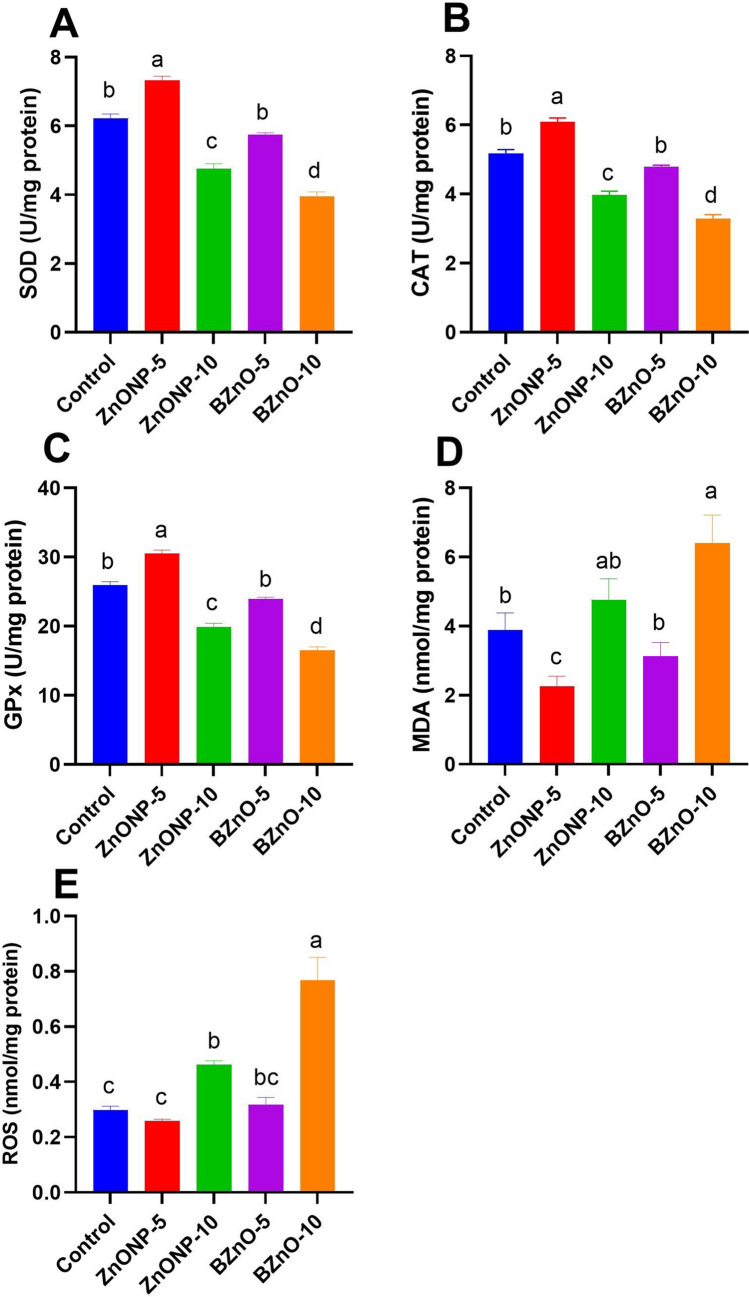
The effects of different doses of ZnONP and BZnO on antioxidant and oxidant levels in brain tissue were evaluated. Specifically, the levels of SOD (superoxide dismutase), CAT (catalase), GPx (glutathione peroxidase), MDA (malondialdehyde), and ROS (reactive oxygen species) were measured. The results are presented as mean ± SEM (standard error of the mean). Statistical analysis revealed that bars labeled with different letters (a, b, c, d) were significantly different from each other at a significance level of *P* < 0.05

**Fig. 3:**
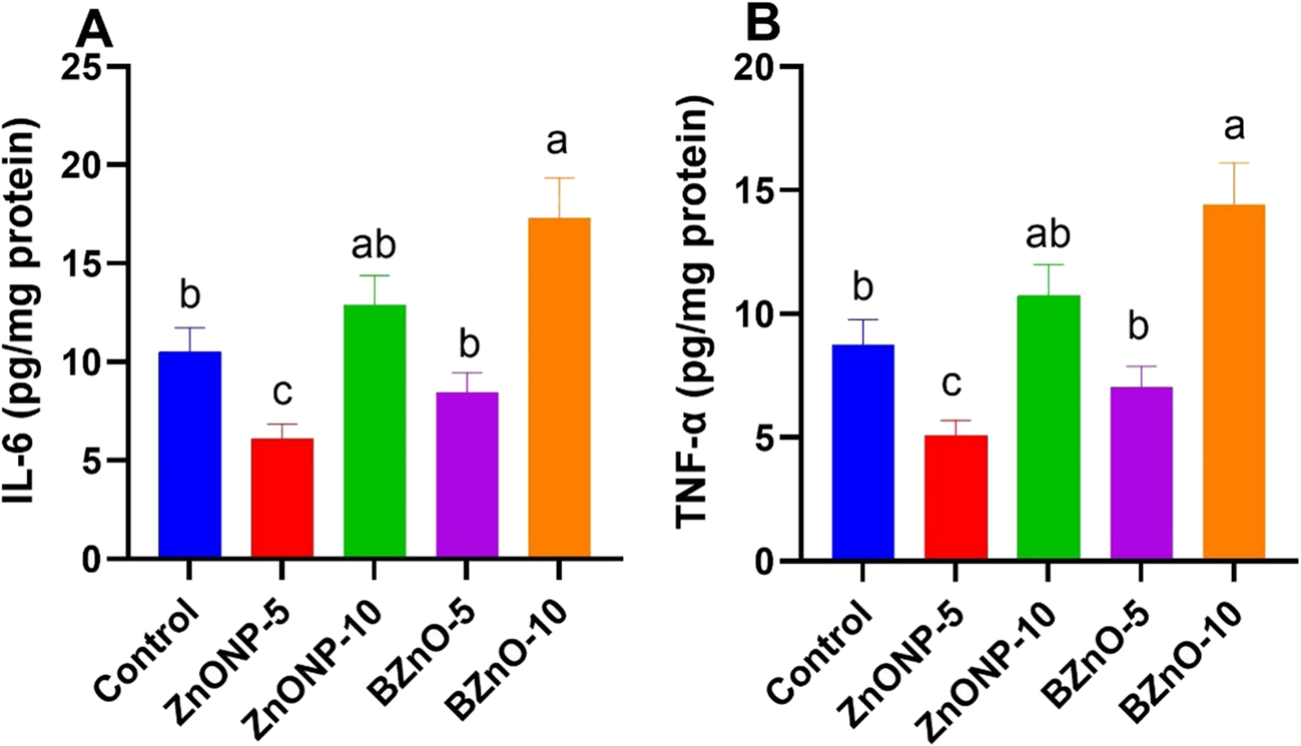
The effects of different doses of ZnONP and BZnO on cytokine and oxidant levels in brain tissue were examined. Precisely, IL-6 (interleukin-6) and TNFα (tumor necrosis factor alpha) were measured. The results are presented as mean ± SEM (standard error of the mean). Statistical analysis revealed that bars labeled with different letters (**a**, **b**, **c**) were significantly different from each other at a significance level of *P* < 0.05

In Fig. 2A, B, and C, a notable elevation in the levels of SOD, CAT, and GPx is evident in the ZnONP-5-treated group compared to the other groups. Similarly, the BZnO-5 group significantly increased compared to the BZnO-10 and ZnONP-10-treated groups. Figure 2D and E reveals a significant decrease in MDA and ROS brain levels in the ZnONP-5 group compared to the other treated groups, although the BZnO-10 group exhibited higher levels. The BZnO-5-treated group demonstrated a significant reduction compared to the ZnONP-10 and control groups. Figure 3A and B shows a notable decrease in the levels of IL-6 and TNF-α in the ZnONP-5 group compared to the other treated groups, particularly the ZnONP-10- and BZnO-10-treated groups. Additionally, the BZnO-5 group exhibited a significant reduction compared to the ZnONP-10 and BZnO-10-treated groups.

### Brain Neurotransmitters

**Fig. 4:**
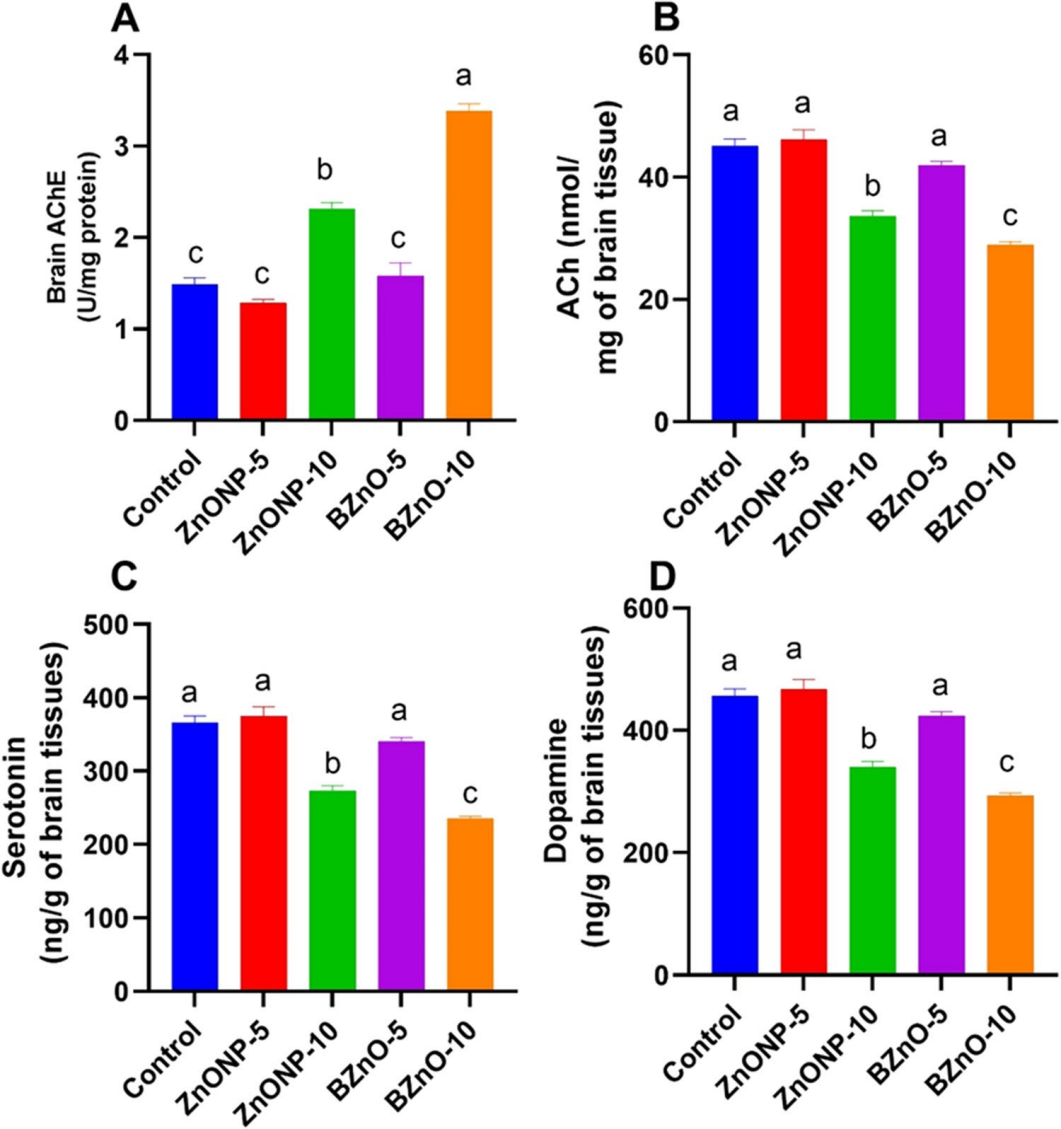
The effects of different doses of ZnONP and BZnO on neurotransmitter levels in brain tissue were investigated. Precisely, the levels of brain AChE (acetylcholinesterase), Ach (acetylcholine), serotonin, and dopamine were measured. The results are presented as mean ± SEM (standard error of the mean). Statistical analysis revealed that bars labeled with different letters (**a**, **b**, **c**) were significantly different from each other at a significance level of *P* < 0.05

Figure 4A, C, and D illustrates a noteworthy rise in the levels of acetylcholine (ACh), serotonin, and dopamine in the ZnONP-5- and BZnO-5-treated groups in comparison to the BZnO-10 and ZnONP-10 groups. However, no significant changes were observed when compared to the control group. In Fig. 4B, the brain level of acetylcholinesterase (AChE) was markedly decreased in the ZnONP-5- and BZnO-5-treated groups as opposed to the BZnO-10 and ZnONP-10 groups. However, the BZnO-10 and ZnONP-10 groups exhibited considerably higher levels of AChE.

### Gene Expression

**Fig. 5:**
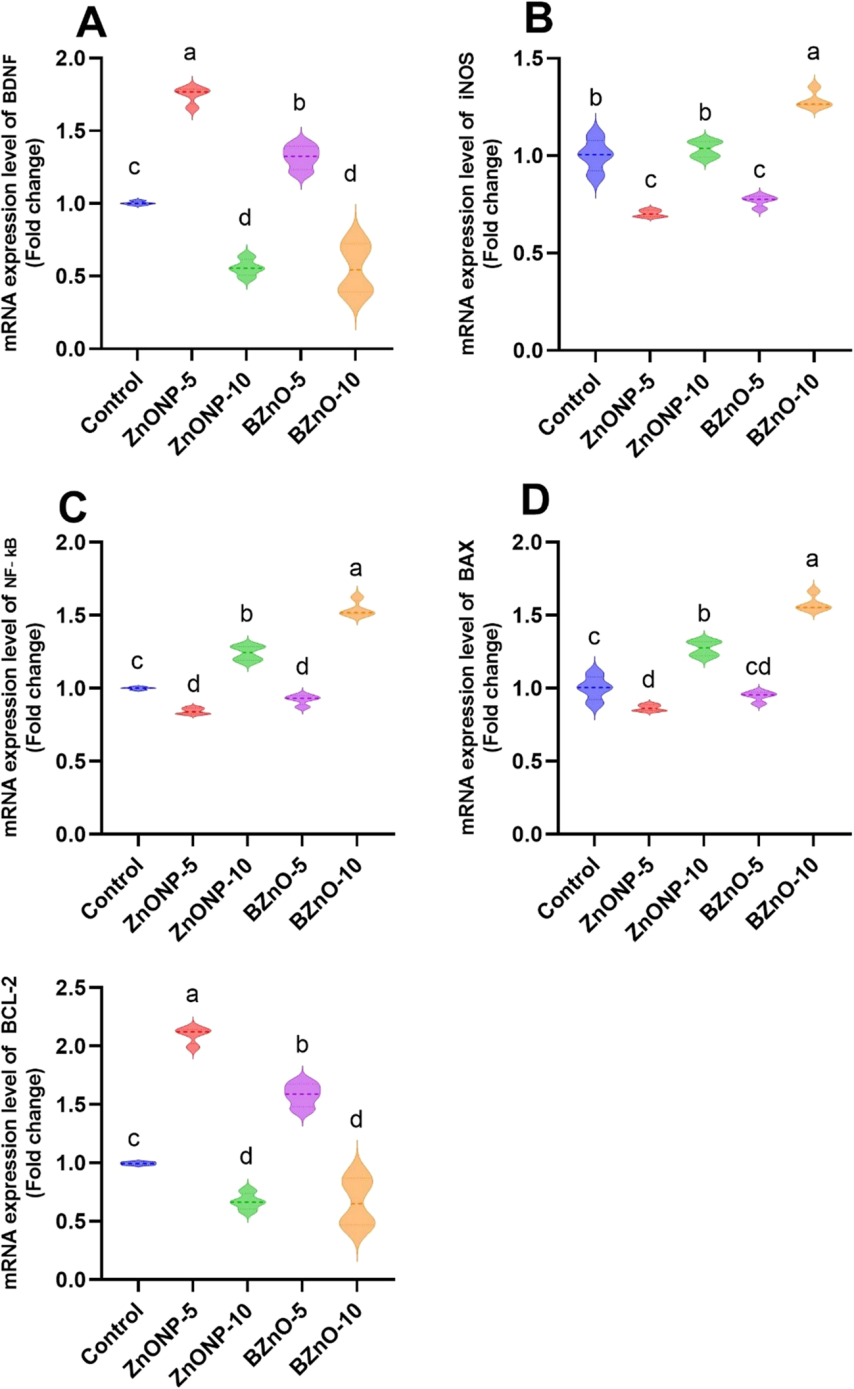
The effects of different doses of ZnONP and BZnO on the mRNA expression of BDNF, iNOS, NF-KB, BAX, and BCL2 were investigated. The results are presented as mean ± SEM (standard error of the mean). Statistical analysis revealed that bars labeled with different letters (**a**, b, **c**, **d**) were significantly different from each other at a significance level of *P* < 0.05

Figure 5 displays the outcomes of gene expression analysis, revealing a noteworthy increase in BDNF mRNA expression in the ZnONP-5-treated group compared to the other treated groups. Similarly, the BZnO-5 group exhibited a substantial upregulation compared to the BZnO-10- and ZnONP-10-treated groups. Additionally, Fig. 5 demonstrates a significant downregulation of iNOS and NF-κB in the ZnONP-5 and BZnO-5 groups compared to the BZnO-10- and ZnONP-10-treated groups. Concerning apoptosis marker genes, there were notable elevations in the mRNA expression of bcl-2 in the ZnONP-5-treated group compared to the other treated groups. Similarly, the BZnO-5 group showed significant upregulation compared to the control, BZnO-10, and ZnONP-10 groups. However, the Bax mRNA expression was significantly downregulated in the ZnONP-5-treated group compared to the other treated groups, with a significant decrease in the BZnO-5 group compared to the control, BZnO-10, and ZnONP-10 groups.

### Histopathological Analysis

**Fig. 6:**
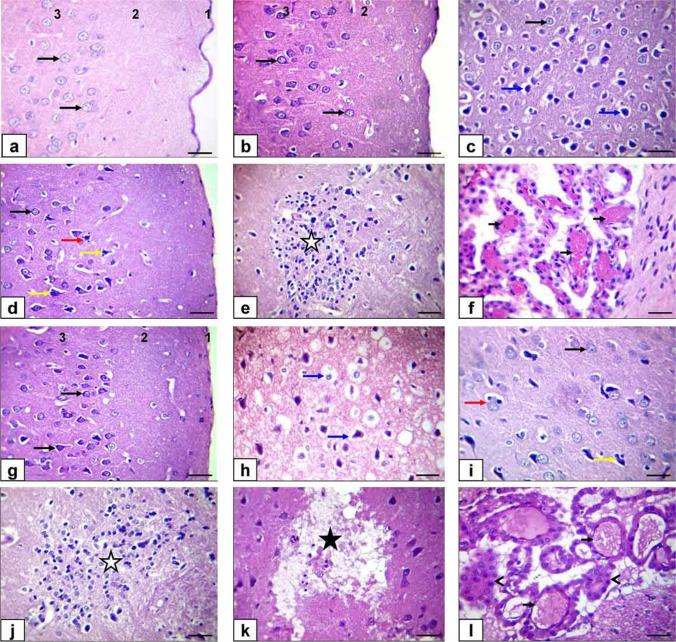
Photomicrograph of the cerebrum sections of rats stained by H&E (bar=50μm). a Control rats. **b** ZnONP-5-treated rats. **c**, **d**, **e**, and (**f**) ZnONP-10-treated rats. g BZnO-5-treated rats. **h**, **i**, **j**, **k**, and l BZnO-10-treated rats. Meninges (1), molecular layer (2), external granular layer (3), normal cortical neuron (black arrows), shrunken, pyknotic, and hyper-eosinophilic cytoplasm with widening of neuropil (blue arrows), satellitosis (red arrow) neuronophagia (yellow arrows), gliosis (white star), polioencephalomalacia (black star), and congestion of choroid plexus vasculature (short black arrows) destruction of choroid plexus epithelium (arrowheads)

The meninges, neurons, and cerebral cortex layer of the control group and ZnONP-5-treated rats displayed a normal histological structure, as observed in Fig. 6a and b. However, in the ZnONP-10-treated rats, the neurons in the cerebral cortex exhibited characteristics such as cell shrinkage, pyknotic nuclei, and hyper-eosinophilic cytoplasm. Additionally, there was evidence of neuropil widening (Fig. 6c), besides satellitosis and neuronophagia of the affected neuron (Fig. 6d), with focal glial cells aggregating in the gray matter (Fig. 6e) and congestion of the vasculature of choroid plexus (Fig. 6f). However, in the BZnO-5-treated rats, the cerebral cortex, hippocampus, and cerebellum showed nearly normal histological structure (Fig. 6g), while in the BZnO-10-treated rats, the cortical neurons showed severe shrunken, pyknotic, and hyper-eosinophilic cytoplasm with the extreme widening of neuropil (Fig. 6h), satellitosis, and neuronophagia of the exaggerated degenerated neurons (Fig. 6i), beside focal gliosis (Fig. 6j), and focal area of polioencephalomalacia with the glial cell reactions also spotted (Fig. 6k) beside congestion of choroid plexus vasculature and destruction of choroid plexus epithelium (Fig. 6l).

**Fig. 7:**
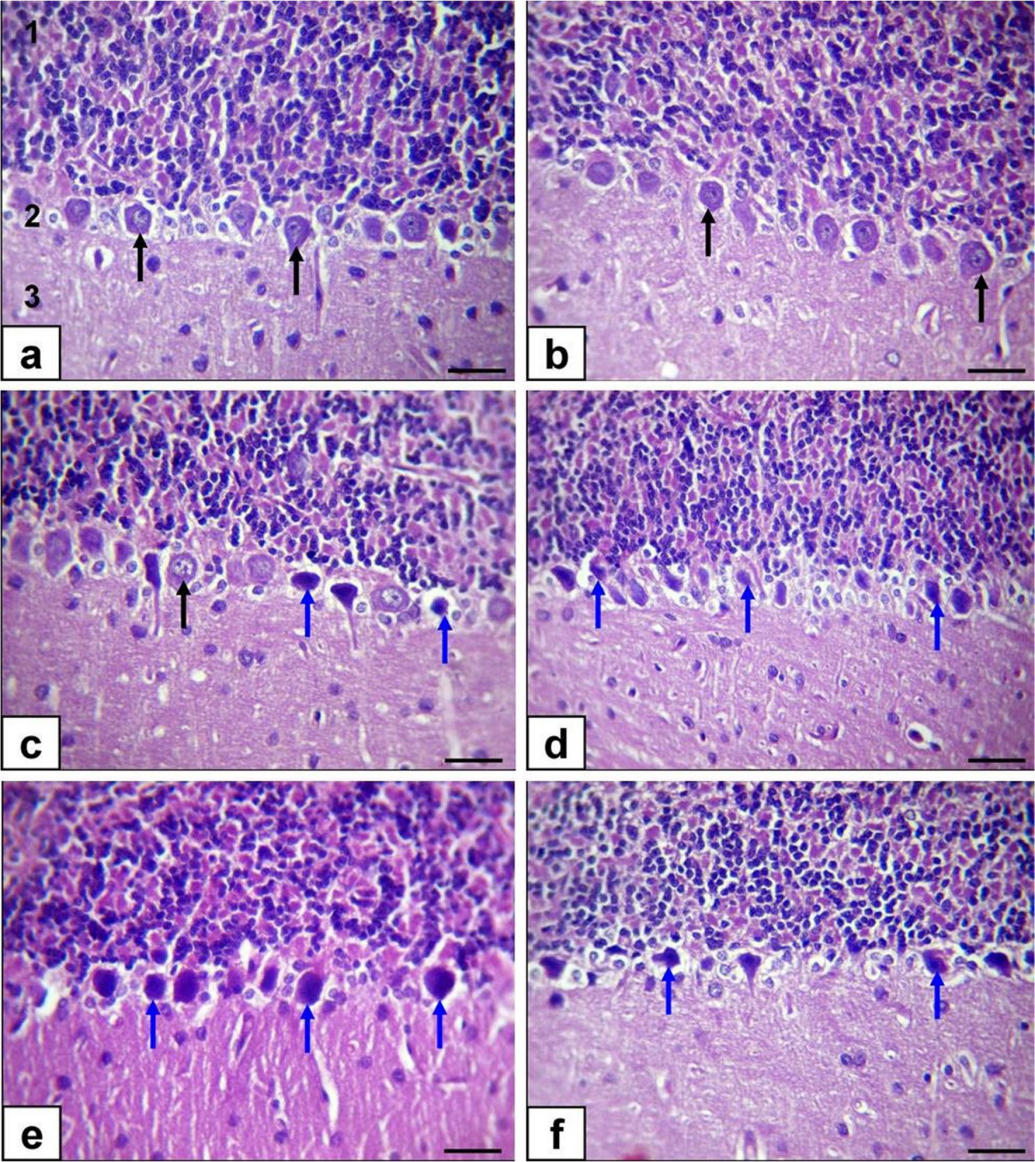
Photomicrographs of the cerebellum sections of rats stained by H&E (bar = 50μm). (**a**) Control rats. (**b**) ZnONP-5-treated rats. (**c**) ZnONP-10-treated rats. (**d**) BZnO-5-treated rats. (**e** and **f**) BZnO-10-treated rats. Granular layer (1), Purkinje cell (2), molecular layer (3), normal Purkinje cell (black arrows), shrinkage, and darkly stained Purkinje cell (blue arrows)

The cerebellar cortex, including the molecular, Purkinje cell, and granular layers, displayed a normal morphology in the control group and ZnONP-5-treated rats, as observed in Fig. 7a and b. However, in the ZnONP-10-treated rats, the cerebellar cortex showed mild abnormalities. These abnormalities included slight shrinkage of cells, cytoplasmic hypereosinophilia (an increase in the staining intensity of cytoplasm), and loss of dendritic arborization in Purkinje cells (Fig. 7c); the same lesion was noticed in BZnO-5-treated rats, which is still moderate in severity (Fig. 7d). In the BZnO-10-treated rats, most Purkinje cells showed shrinkage, darkly stained, loss of dendritic arborization, and depletion in number (Fig. 7e and f); besides, few numbers of granular cell layer were recorded in few rats.

### Immunohistochemistry of Caspase-3 Protein Expression

**Fig. 8:**
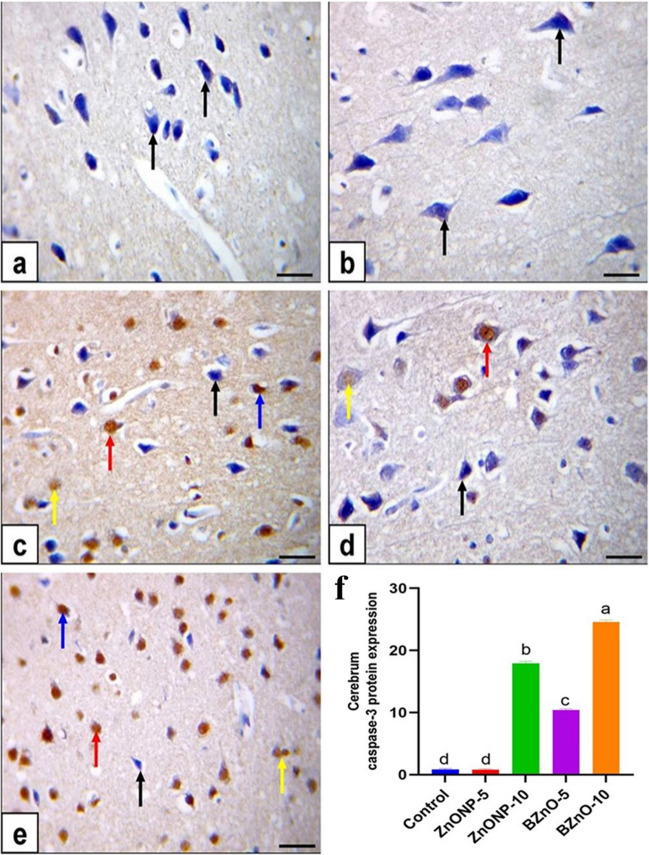
Immunohistochemical staining of the cerebrum was performed to evaluate the expression of caspase-3 protein, as shown in figure. The scale bar represents 50μm. In the control group (**a**) and ZnONP-5-treated rats (**b**), the staining displayed negative immunostaining for caspase-3 in cortical neurons, indicated by black arrows. In the ZnONP-10-treated rats (**c**), moderate immunostaining was observed, represented by red arrows. In the BZnO-5-treated rats (**d**), most cortical neurons exhibited negative staining (black arrows), while a few neurons showed weak immunostaining (yellow arrows). In the BZnO-10-treated rats (**e**), intense immunostaining for caspase-3 was observed in cortical neurons, denoted by blue arrows. (**f**) Area % of caspase-3 protein immunostaining positive cell. All the values are expressed as mean ± standard error of mean. Statistical analysis revealed that bars labeled with different letters (a, b, c, d) were significantly different from each other at a significance level of *P*< 0.0001

**Fig. 9:**
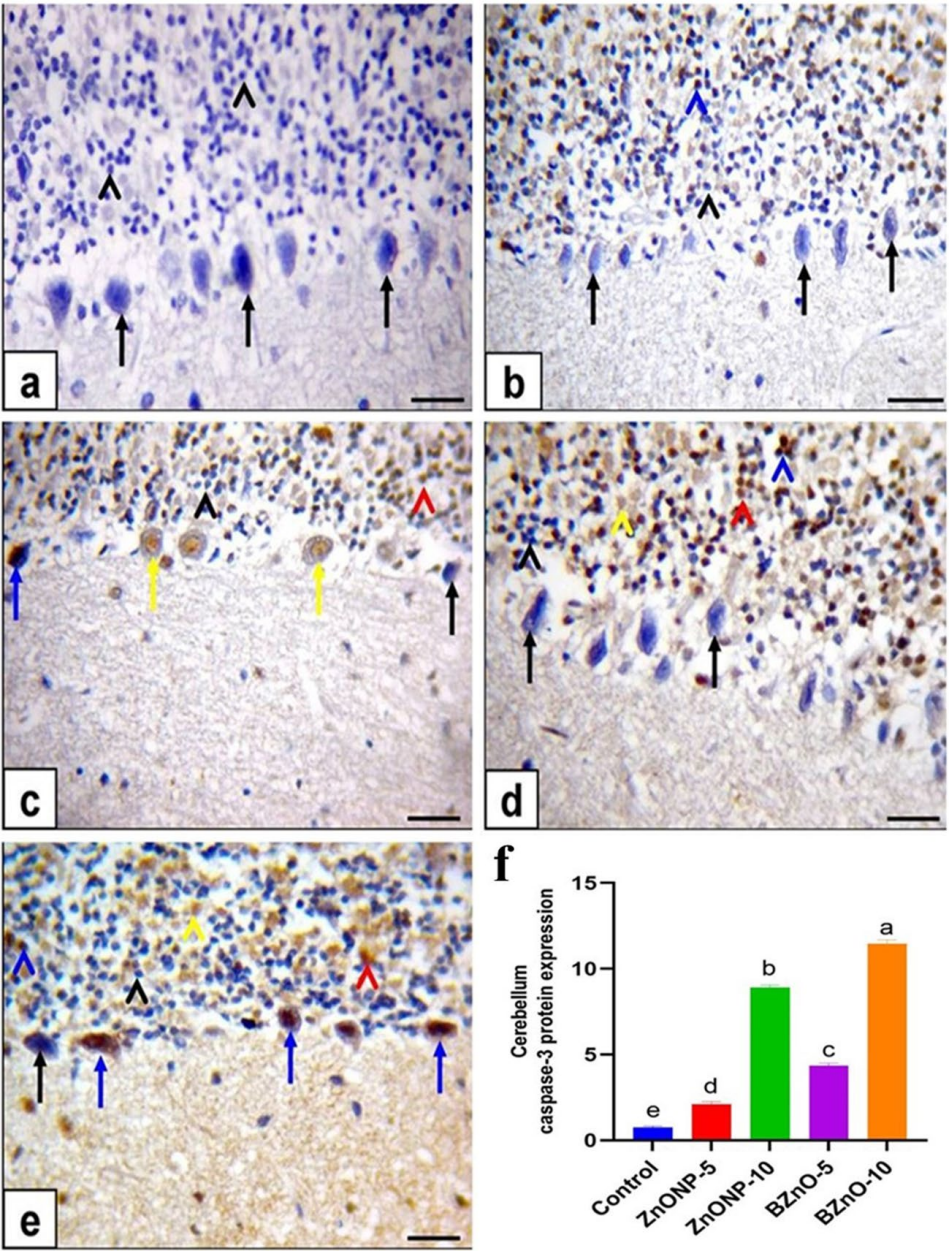
Immunohistochemical staining of the cerebellum was performed to assess the expression of caspase-3 protein, with a scale bar of 50μm. In the control rats (a), both neurons and granular cells exhibited negative immunostaining for caspase-3, indicated by black arrows and arrowheads. In the ZnONP-5-treated rats (**b**), the Purkinje cells displayed negative brown staining (black arrow), while the granular cells showed intense immunopositive brown staining (blue arrow). In the ZnONP-10-treated rats (**c**), both Purkinje cells and granular cells exhibited moderate to weak immunopositive brown staining, represented by red arrows and arrowheads. In the BZnO-5-treated rats (**d**), the Purkinje cells showed negative brown staining (black arrow), while the granular cells displayed moderate immunopositive brown staining (yellow arrow). Moreover, in the BZnO-10-treated rats (**e**), both Purkinje cells and granular cells exhibited moderate immunopositive solid brown staining, denoted by red arrows and arrowheads. (**f**) Area % of caspase-3 protein immunostaining positive cell. All the values were expressed as mean ± standard error of mean. Statistical analysis revealed that bars labeled with different letters (a, b, c, d, e) were significantly different from each other at a significance level of *P*< 0.0001

The cerebral cortical neurons in the control group and ZnONP-5-treated rats displayed immune-negative brown staining for caspase-3, as shown in Fig. 8a and b. However, in the ZnONP-10-treated rats, moderate immune-positive solid brown staining for caspase-3 was observed, as depicted in Fig. 8c. On the other hand, in the BZnO-5-treated rats, most cerebral cortical neurons exhibited negative brown staining for caspase-3. In contrast, a few neurons showed weak immune-positive brown staining, as seen in Fig. 8d. In contrast, the cerebral cortical neurons in the BZnO-10-treated rats exhibited intense immune-positive brown staining for caspase-3 (Fig. 8e). The cerebellum, including Purkinje cells and granular cells, was subjected to immunohistochemical staining. Purkinje and granular cells exhibited immune-negative brown staining in the control rats, as depicted in Fig. 9a.

Conversely, in ZnONP-5-treated rats, Purkinje cells showed negative brown staining, while granular cells displayed intense immune-positive brown staining, as shown in Fig. 9b. In ZnONP-10-treated rats, Purkinje and granular cells exhibited moderate to weak immune-positive brown staining, as seen in Fig. 9c. In the BZnO-5-treated rats, Purkinje cells showed negative brown staining, while granular cells displayed moderate immune-positive brown staining, as shown in Fig. 9d. Furthermore, in the BZnO-10-treated rats, Purkinje and granular cells exhibited moderate immune-positive solid brown staining, as depicted in Fig. 9e. The average percentage of cells expressing caspase-3 protein through immunostaining in the cerebrum and cerebellum showed a notable reduction in ZnONP-5, followed by BZnO-5 rats. Conversely, a significant increase was observed in ZnONP-10 and BZnO-10 rats compared to the control rats.

## Discussion

Previous research has documented a range of advantageous effects associated with ZnONPs. However, its adverse effects have been increasingly studied [37], and the exact dose and safety of ZnONPs on cognitive function the precise mechanisms and consequences of ZnONPs have not been fully elucidated. To answer the question, this study evaluated the impact of ZnONPs on brain function and behavior with different doses compared to its bulk form.

The current study revealed that the ZnONP at 5mg/kg showed improvement in the general behavioral patterns, including feeding, drinking, standing, movement, and investigation, whereas lying, licking, and scratching behaviors decreased than the bulk form with a similar dose. Furthermore, it also showed the best locomotor activity and the least fearfulness in the open field test. Also, ZnONP at 5mg/bwt treated rats were the most anxiolytic, indicated by shorter entry time to open arms, multiple entries in open arms, and longer presence time in the elevated plus maze. The learning ability of rats measured by the Morris water maze indicated that ZnONP with 5mg/kg treated rats had the shortest time to locate the hidden platform and a longer time of presence in the target quadrant. On the contrary, ZnONP with 10 mg/kg treated rats showed decreased general behaviors, locomotor activity, and learning ability. These indicated that ZnONPs had a toxic effect at higher doses. However, the worst impact was for the BZnO-10 group on behavioral patterns, locomotor activity, and learning ability.

The anxiolytic effect of ZnONPs at a dosage of 5 mg/kg may be attributed to the involvement of zinc in anxiety-related neurochemical systems. Zinc plays a role in the co-release with glutamate in presynaptic spaces, which exerts an inhibitory neuromodulatory effect on glutamate signaling [19]. Glutamate was found to be an essential element in anxiety and anxiogenic behavior, so blocking of NMDA glutamate receptors by zinc could elicit an anxiolytic effect [38]. Additionally, zinc has been found to promote the release of gamma-aminobutyric acid (GABA) from interneurons in the hippocampus. This enhances GABA’s inhibitory effect, thereby reducing glutamate’s presynaptic release [39].

Consequently, maintaining a balance between the inhibitory effects of GABA receptors and the excitatory effects of glutamate receptors could play a crucial role in regulating the behavioral and physiological responses related to anxiety [40]. Similar to our study, Torabi et al. [41] stated that the better anxiolytic and locomotive effect of ZnONPs 5mg/kg than its conventional form. These could be due to the greater mobility of ZnONPs and their uptake across biological membranes, increasing their interaction with biological tissues [42]. ZnO nanoparticles have a larger surface area than the conventional form, resulting in increased reactivity due to the higher number of exposed reactive groups [43]. These findings suggest that even the lowest dose of ZnONPs is sufficient to release an amount of zinc that can interact with the corresponding receptor structures. However, higher doses only lead to saturation of serum zinc levels and reduced anxiolytic effects. Additionally, the performance improvement observed in both the control group and the rats treated with BZnO at 5 mg/kg may be attributed to their cognitive flexibility [44].

Earlier studies have demonstrated that the administration of nanoparticles to rats or mice, either systemically or intracerebrally, can result in cognitive function alterations and neurotoxicity. However, the extent of these effects depends on factors such as the type of nanoparticles used, the dosage administered, and the duration of exposure [45]. For example, Goma et al. [46] reported neurobehavioral toxicity for CuO-NPs.

The central nervous system (CNS) is sensitive to interactions with ions in physiological and pathological conditions [47]. For example, ZnONPs could leach zinc ions that diffuse to cells and/or organs like CNS [48]. CNS is protected by the blood-brain barrier (BBB), a critical barrier for regulating the transport of substances into the brain. ZnONPs were reported to cause alteration in the integrity and permeability of the BBB due to inflammation [49]. Furthermore, previous reports stated that ZnO dissolution is enhanced in the case of nanoparticles [50], which could have a role in ZnONPs toxicity [51].

Neurodegenerative illnesses have been linked to the accumulation of metals like copper (Cu), aluminum (Al), and zinc (Zn). These conditions are characterized by the loss of function and damage to cells in the affected areas [52]. Moreover, studies have reported that ZnONPs can disrupt zinc homeostasis in synapses. This disruption can result in hyperactive long-term potentiation and inadequate depotentiation, potentially affecting the normal functioning of neuronal synapses [53]. Furthermore, ZnONPs have been observed to induce intracellular calcium accumulation by enhancing sodium influx and increasing neuronal excitability. The concentration of intracellular calcium is crucial for long-term potentiation and long-term depotentiation in the induction and maintenance phases. These findings suggest that the toxicity of ZnONPs depends on the dosage rather than the size of the nanoparticles [54].

Like our study, De Souza et al. [55] indicated anxiogenic behavior in male mice subjected to ZnONPs by 300 mg/kg intraperitoneally for 5 days, which could be due to Zn accumulation in the brain or the impairment of anxiety-related neurological circuit function that can be attributed to the ability of these nanoparticles to interfere with normal neuronal activity. Torabi et al. [41] showed that ZnONP exposure by 20 mg/kg induced anxiety-like behaviors and reduced locomotor activity in rats after an intraperitoneal injection. However, a study demonstrated that administering ZnONPs with a size range of 20–30 nm at a dosage of 25 mg/kg via intraperitoneal injection for ten consecutive days resulted in minor alterations in the exploratory behavior of rats. However, no significant differences were observed in the anxiety index compared to the control group. These findings suggest that the effects of ZnONP exposure depend on factors such as the species being studied, the route and duration of exposure, and the concentrations or doses administered [56]. Another study by Xie et al. [24] showed that exposure to 5.6 mg/kg ZnONPs in mice damaged spatial cognition.

Furthermore, Han et al. [53] reported disturbance in the spatial cognition by ZnONPs within 20–80 nm injected intraperitoneally in rats by 4 mg/kg biweekly for 8 weeks that could be due to alteration of synaptic plasticity, enhanced long-term potentiation, and rigid cognitive flexibility due to repeated exposure. Zhao et al. [23] also reported that ZnONPs could potentially influence neurotransmitter disorders in the central nervous system for their regulatory role in the physiological functions of neurons. However, Chuang et al. [57] studied acute exposure to ZnONPs by 5 and 10 mg/kg but reported no significant changes in spatial cognition, learning ability, or anxiety. In contrast, our study, similar to previous research, found that the administration of 10 mg/kg of ZnONPs resulted in a decrease in the time spent in the open arms, indicating increased anxiety-like behavior.

As a cofactor for many enzymes, zinc is essential for many biological processes, including antioxidant defense mechanisms. This research looked into zinc oxide nanoparticles’ antioxidant and anti-inflammatory properties. Antioxidant enzymes such as glutathione peroxidase (GPx), catalase (CAT), and superoxide dismutase (SOD) were found to have significantly increased levels. Furthermore, compared to the other treatment groups, those given ZnONP-5mg/kg showed substantially lower levels of MDA (a marker of oxidative stress) and the pro-inflammatory cytokines IL-6 and TNF-α. These findings suggest that ZnONPs possess antioxidant and anti-inflammatory properties. These results line up with what has been found before, suggesting the beneficial effects of zinc nanoparticles in modulating antioxidant and anti-inflammatory responses [58, 59]; according to their results, ZnONPs were found to reduce oxidative stress in testicular tissue by decreasing levels of MDA and increasing levels of antioxidants such as SOD, C.A.T., GPx, and GR. As well as, [60] reported that ZnONPs have a protective effect against cognitive impairment and anxiety caused by aging, mitigating associated oxidative damage. Zinc is essential for functioning antioxidant enzymes like SOD and is known to protect sulfhydryl groups by displacing other metals from catalytic sites. Therefore, ZnONPs can help preserve the integrity of cell membranes by shielding them from oxidative injury, increasing antioxidant levels, and reducing free radicals [61].

Compared to other tissues, the brain has a lower level of antioxidant enzymes, higher oxygen consumption, and more lipids, making it more susceptible to oxidative damage. Reactive oxygen species produced during aerobic metabolism mainly contribute to age-related neuronal damage [62]. The presence of ROS and their byproducts can have detrimental effects on DNA, proteins, and lipids. This can result in modifications to enzyme systems and trigger cell death pathways [60]. Our result revealed that The ZnONP-5 mg/kg treated group exhibited a notable reduction in brain levels of ROS related to the other treated groups. However, the BZnO-10 mg/kg group had higher levels of ROS. Conversely, in the BZnO-5 mg/kg treated group, a significant reduction in reactive oxygen species (ROS) levels was observed compared to the ZnONP-10 mg/kg and control groups.

The administration of ZnONPs to aged rats improved their oxidative status, bringing it closer to the levels observed in adult control rats. This indicates that zinc has a benefit in reducing oxidative stress [63]. Previous studies have shown that very low and very high zinc levels are correlated with increased oxidative injury. Nonetheless, moderate levels have been found to have a neuroprotective effect. This finding aligns with the current study, where an average dose of ZnONPs was utilized, providing a plausible explanation for the positive results observed with zinc supplementation [60].

The ZnONP-5 mg/kg group substantially reduced IL-6 and TNF-α levels compared to the other experimental groups, specifically the ZnONP-10 mg/kg and BZnO-10 mg/kg groups. Moreover, the BZnO-5 mg/kg group exhibited substantially lower levels of IL-6 and TNF-α than the ZnONP-10 mg/kg and BZnO-10 mg/kg treated groups. These results align with what other research has shown about the anti-inflammatory effects of zinc nanoparticles at lower doses [64]. For instance, a previous study reported that ZnONPs possess potent antioxidant activity, which can scavenge 45.46% of DPPH at a concentration of 1 mg/ml. Additionally, the study showed that ZnONPs dose-dependently suppressed the mRNA and protein expressions of vital inflammatory markers such as iNOS, COX-2, IL-1β, IL-6, and TNF-α. These findings collectively suggest that ZnONPs possess effective anti-inflammatory properties.

AChE is an essential enzyme involved in cholinergic neurotransmission, responsible for breaking down acetylcholine (ACh) to terminate synaptic transmission. Changes in AChE activity can directly impact ACh levels. Altered AChE activity has been associated with neurological disorders like Alzheimer’s disease [65]. Our study observed a substantial reduction in AChE activity in the brains of rats treated with ZnONPs. This suppressed AChE activity can hinder the degradation of ACh in the synaptic cleft, potentially leading to increased ACh accumulation. This finding supports our results regarding elevated ACh levels in the brain and suggests that the suppression of AChE activity is another potential mechanism contributing to this effect; our impact was also strengthened by [66] in which they found that the reduced activity of AChE in aged rats could be ascribed to the formation of free radicals during the aging process, which disrupts the balance between prooxidants and antioxidants in the brain. This could explain the beneficial effect of ZnONPs, which act as antioxidants, on the activity of AChE in aged rats. This effect brings the AChE activity levels closer to those observed in the control adult group [60].

Dopamine is an ample neurotransmitter in the CNS and is crucial in various cognitive functions [67]. Catecholamines, including dopamine, are essential for learning and memory [68]. Similarly, serotonin levels have been linked to learning and memory consolidation [69]. In this investigation, only animals displayed high BZnO-10 mg/kg doses, and ZnONP-10 mg/kg exhibited reduced neurotransmitter levels (dopamine and serotonin) in the brain tissue. Indeed, no substantial differences were observed in the BZnO-5 mg/kg and ZnONP-5 mg/kg treated groups compared to the control groups. This suggests that higher doses of zinc nanoparticles may be harmful, as the deposit of zinc ions in post-synaptic neurons can result in toxicity to nerve cells [70]

Brain-derived neurotrophic factor (BDNF) is an essential member of the neurotrophin family and plays a significant role in promoting neuron growth, survival, and differentiation. Research conducted on live organisms has demonstrated that BDNF enhances cholinergic neurotransmission in the central nervous system, influences neuronal plasticity, and protects against neurodegenerative disorders. BDNF achieves this protective effect by increasing the activity of endogenous neuroprotective strategies, including antioxidant pathways [71]. The gene expression analysis revealed a significant increase in the mRNA expression of BDNF in the ZnONP-5 mg/kg treated group linked to the other treatment groups. Similarly, the BZnO-5 mg/kg group showed a considerable upregulation of BDNF mRNA expression compared to the BZnO-10 mg/kg and ZnONP-10 mg/kg treated groups. Consistent with prior studies, these results show that zinc nanoparticles have them, particularly at lower doses, to enhance the expression of BDNF, which is associated with neuroprotection and neuronal plasticity [72]. Their study proved that ZnONP treatment leads to increased BDNF gene expression, counteracting the hippocampal neurotoxicity induced by type 2 diabetes in rats.

The administration of monosodium glutamate and ZnO nanoparticles significantly increased brain tissue levels of BDNF in rats [73]. This suggests that zinc has a positive influence on reducing oxidative stress. Previous studies have demonstrated that both zinc deficiency and excessive zinc levels are associated with elevated oxidative stress. However, moderate levels of zinc have been found to have neuroprotective effects. The current findings explain the beneficial effects observed in this study, where an appropriate dose of ZnONPs was administered [74]. Therefore, it can be suggested that the neuroprotection offered by ZnONPs may be directly related to their antioxidant pursuit.

Our findings were consistent with [75] study, in which they showed a noteworthy reduction in the expression levels of iNOS (inducible nitric oxide synthase) and NF-κB (nuclear factor-kappa B) in both the ZnONP-5 mg/kg and BZnO-5 mg/kg groups associated to the BZnO-10 mg/kg and ZnONP-10 mg/kg groups. These consistent results provide additional evidence supporting the potential anti-inflammatory properties of lower doses of zinc nanoparticles. Previous studies, such as the one conducted by Kim [75], have reported that ZnO nanoparticles exhibit anti-inflammatory consequences by stopping the boost in inflammatory cytokines and inhibiting the activation of NF-κB. Activation of the NF-κB pathway fronts to the induction of various inflammatory cytokines and chemokines. Once activated, NF-κB translocates to the nucleus and binds to specific DNA sequences, thereby promoting the production of inflammatory mediators such as cyclooxygenase-2, inducible nitric oxide synthase, tumor necrosis factor-α, interleukin-1β, and interleukin-6. The ability of zinc nanoparticles to inhibit NF-κB activation and suppress the creation of these inflammatory mediators suggests their potential as anti-inflammatory agents [64, 76]

The ZnONP-5 mg/kg group showed a considerable enhancement in the anti-apoptotic gene bcl-2 expression, while a substantial reduction in the expression of the pro-apoptotic gene Bax was observed. The BZnO-5 mg/kg bwt group also exhibited similar gene expression patterns compared to the control, BZnO-10 mg/kg bwt, and ZnONP-10 mg/kg bwt groups; these findings aligned with Tuzcu et al. [77]. They suggested that ZnONPs can mitigate the effects of cisplatin by preventing apoptosis. This is achieved by stabilizing biomembranes with zinc and its indirect antioxidant activity. In the same context, the immunohistochemistry of caspase-3 protein expression strengthens our finding concerning apoptosis, as reported by Barakat et al. [78]. They said that the treatment with ZnONPs demonstrated a protective effect on the kidneys against the toxic effects of cisplatin by its antioxidant, anti-inflammatory, and antiapoptotic properties; zinc nanoparticles exhibit a range of beneficial effects. These properties allow zinc nanoparticles to counteract oxidative stress, reduce inflammation, and prevent cell death.

The histological findings are consistent with the behavioral, molecular, and biochemical data. The histological examination revealed that the control and ZnONP-5 mg/kg treated rats had normal meninges, neurons, and cerebral cortex structures. However, ZnONP-10 mg/kg treated rats accompanied considerable abnormalities such as shrinkage, pyknosis, hyper-eosinophilic cytoplasm, widening of neuropil, satellitosis, neuronophagia, glial cell aggregates, and vascular congestion in the choroid plexus. The rats treated with BZnO-5 mg/kg exhibited cerebral cortex, hippocampus, and cerebellum with histological structures that were almost normal. On the other hand, the rats treated with BZnO-10 mg/kg showed significant shrinkage of cortical neurons, loss of dendritic arborization in Purkinje cells, gliosis (an abnormal increase in glial cells), and areas of tissue damage.

Additionally, there were a few instances of a reduced number of granular cells in the cerebellar cortex. The histopathological analysis revealed no changes in the rats treated with ZnONPs linked to the control groups. These results align with previous studies conducted by Wang et al. [79], Zheng et al. [80], and Hamza et al. [73], which furthermore demonstrated the lack of toxic effects of ZnONPs on brain tissue. In contrast, Win-Shwe et al. reported poisonous results of ZnONPs on the CNS, including cytotoxicity, inflammation, and induction of oxidative stress leading to neurodegeneration [81]. In contrast, Gantedi et al. conducted a study and reported that a dose of 5 mg/kg of ZnONPs did not lead to noticeable alterations in the brain histology of rats after one month of treatment [82]. It is essential for both laboratory and clinical studies to carefully consider the duration, dosage, and route of administration when evaluating the cellular toxicity of ZnONPs in different organs, including nerve tissue. These factors significantly contribute to determining the specific characteristics and potential toxicity associated with the nanoparticles.

## Conclusion

This study pioneered investigating the effects of different forms of zinc salt, explicitly comparing the impact of zinc oxide nanoparticles (ZnONPs) and bulk zinc on various aspects of rat neurobehavior, antioxidant levels, gene expression, and brain histopathology. The results indicated that a lower dosage of 5 mg/kg ZnONPs had a beneficial effect on multiple aspects of behavior and brain function, including memory, recognition, learning ability, and neurotransmitter levels. A higher dosage of 10 mg/kg could be detrimental. Furthermore, the study emphasizes the necessity for additional investigations to ascertain the safety profile of ZnONPs on cognitive function and brain health fully.

### Supplementary information

ESM 1 (DOCX 17 kb)

## Data Availability

No datasets were generated or analysed during the current study.
