# Supplementary material for: Examining the Influence of Zinc Oxide Nanoparticles and Bulk Zinc Oxide on Rat Brain Functions: a Comprehensive Neurobehavioral, Antioxidant, Gene Expression, and Histopathological Investigation
Source: Biol Trace Elem Res. 2024 Jan 8;202(10):4654–73. doi: 10.1007/s12011-023-04043-x (PMC11339107; doi:10.1007/s12011-023-04043-x)
Supplement: Supplementary file 1 — (DOCX 17 kb) [file 12011_2023_4043_MOESM1_ESM.docx]

**Table 1S. Ingredients of the basal diet**

| g/kg diet | Ingredients |
| --- | --- |
| 529.5 | Corn flour |
| 200 | Casein |
| 100 | Sucrose |
| 70 | Soybean oil |
| 50 | Cellulose |
| 35 | Mineral mix |
| 10 | Vitamin mix |
| 3 | L-cystine |
| 2.5 | Choline |

**Table 2S.** Primers for gene expression by RT-PCR.

| Gene | Direction | Primer sequence | Accession number |
| --- | --- | --- | --- |
| *Bax* | Sense | GGCGAATTGGCGATGAACTG | NM_017059.2 |
|  | Antisense | ATGGTTCTGATCAGCTCGGG |  |
| *Bcl-2* | Sense | GATTGTGGCCTTCTTTGAGT | NM_016993.1 |
|  | Antisense | ATAGTTCCACAAAGGCATCC |  |
| *NF‐κB* | Sense | CTCCGC GGG CAG CAT CC | NM_001415012 |
|  | Antisense | AGC CGC ACA GCA TTCAGG TCG TAG |  |
| *iNOS* | Sense | CAG GAC CAC ACC CCC TAGGA | X59949 |
|  | Antisense | AGC CAC ATA CCG AGC CAT GC |  |
| *BDNF* | Sense | CAGGGGCATAGACAAAAG | NM_012513.4 |
|  | Antisense | CTTCCCCTTTTAATGGTC |  |
| *GAPDH* | Sense | TCAAGAAGGTGGTGAAGCAG | NM_017008.4 |
|  | Antisense | AGGTGGAAGAATGGGAGTTG |  |

Bax, Bcl-2-associated X protein. Bcl-2, B-cell lymphoma 2. BDNF, Brain-derived neurotrophic factor. GAPDH, glyceraldehyde-3-phosphate dehydrogenase. iNOS, Nitric oxide synthase. *NF‐κB*, nuclear factor kappa B
